# PCR Detection of Microbial Pathogens

**DOI:** 10.3201/eid1908.130528

**Published:** 2013-08

**Authors:** Deborah F. Talkington

**Affiliations:** Centers for Disease Control and Prevention, Atlanta, Georgia, USA

**Keywords:** PCR, real-time PCR, microbial pathogens, molecular diagnostics

This book covers general challenges of introducing primarily noncommercial PCRs and specific procedures into the laboratory, including sample treatment, extraction protocols, quality and quality assurance, and internal and external laboratory processes. The chapters on specific pathogens illustrate principles that could be applied in many diagnostic laboratories.

The editor’s preface to this book is helpful in framing approaches to PCR pathogen detection methods. The focus is primarily on detection of bacterial pathogens, with the exception of *Pneumocystis *spp., and the case is made for using less expensive noncommercial strategies that enable more flexibility and customization. The book addresses the many parameters of nucleic acid preparation, buffer choice, primer construction, inhibition, cycling parameters, detection, and statistical analysis.

The ≈300 pages of text are divided in 21 chapters, of which the first 3 cover concepts of importance to all clinical laboratories using PCRs. The third chapter, which covers quality and quality assurance, is particularly comprehensive in its treatment of internal and external laboratory process and PCR controls. This chapter covers a variety of concepts, from Westguard rules for investigations of systematic and other errors, to proficiency testing, and includes many useful tables. Of importance to clinical laboratories and epidemiologic investigations alike, the authors make an essential point that up to 75% of errors in the testing process can be attributed to improper sample collection and transport of specimens, areas that often get less attention than assay quality control. The fourth chapter covers preanalytical and extraction protocols specifically for molecular detection of pathogens in whole blood, which is a particularly challenging specimen.

The remaining chapters cover a mixture of mostly real-time and some conventional PCRs targeting specific pathogens (sometimes by multiplex approaches), and 1 chapter describes a loop-mediated isothermal amplification method for detection of *Campylobacter *spp. The pathogens and techniques covered represent a good survey of approaches and vendor equipment choices. The quality of the chapters in this book varies widely, and some repetitive information is included. Overall, this book would be of interest to those involved in PCR principles and laboratory quality control. It contains examples of successful noncommercial diagnostic PCRs. If your pathogen(s) of interest are covered, it is an added bonus.

